# The Evolving Incidence of Hepatitis C Virus Infection in Italy

**DOI:** 10.5812/hepatmon.820

**Published:** 2012-02-29

**Authors:** Rinaldo Pellicano, Sharmila Fagoonee

**Affiliations:** 1 Department of Gastroenterology and Hepatology, Molinette Hospital, Turin, Italy; 2 Molecular Biotechnology Center, University of Turin, Turin, Italy

**Keywords:** Hepatitis C, Liver Cirrhosis, pidemiology

Dear Editor,

The epidemiology of chronic liver diseases (CLD) in Italy is changing. This is due to the decreasing rate of viral hepatitis [1] and the increased number of steatosis and nonalcoholic steatohepatitis cases, which are emerging as a new epidemic with a wide spectrum of metabolic disorders [2]. This scenario reflects what has occurred in Italy over the last few decades, as the vaccination program against the Hepatitis B virus (HBV), mandatory since the 1991, has helped to achieve complete immunization of newborns, adolescents and young adults [3]. Simultaneously, the epidemiology of the Hepatitis C virus (HCV) has evolved, due to factors such as increased blood transfusion safety, improvements in healthcare conditions, continuing expansion of intravenous drug use (IDU) and immigration from endemic areas. The lack of a prophylactic vaccine or universally active therapy has made the prevention of this chronic infection extremely important. Identification of infected persons and of the risk factors associated with acquiring a HCV infection may allow the development of strategies to reduce its incidence and control the resulting epidemic [4].

The prevalence of the HCV in Europe is nearly 1%, but this varies geographically along a north-south gradient, ranging from approximately 0.5% in Northern countries to 2% in Mediterranean areas [5]. In Northern Italy, the Dionysos study collected data regarding 6,917 inhabitants of two towns (Campogalliano and Cormons). The overall prevalence of positivity to the HCV antibody was found to be 3.2%. When the prevalence was analysed according to different age groups, it was relatively low ( < 1%) up to 40 years, but the rate rose sharply thereafter, to reach a value of 10% in subjects older than 60 years [6]. This suggested a cohort effect. In a rural area of Central Italy, Raffaele et al. carried out a prevalence study, which included 344 subjects selected by random sampling among 3,308 inhabitants older than 16 years. The inferred HCV positivity prevalence rate was 22.4% with an increased trend particularly evident as a result of ageing [7]. In a Southern Italian town, Cozzolongo et al. found a seroprevalence for anti-HCV positivity of 2.6%; this prevalence increased from 1% in subjects aged < 30 years to 7.7% in those > 70 years [8]. The incidence of HCV infection is very difficult to estimate accurately because many patients with acute HCV infection are asymptomatic, and thus, do not present themselves for diagnosis [4]. Data from the US suggests that the annual incidence of HCV infection fell from 230,000 new cases per year in the late 1980s to approximately 35,000 new cases per year in the 1990s [4]. In Northern Italy, Mazzeo et al. carried out a study on the ten year incidence of HCV infection in a representative cohort of 1,646 adults from the general population of two Apennine hill towns. The incidence was 50.3 cases per 100,000 inhabitants/year while 16.9% of anti-HCV positive subjects spontaneously cleared the virus while 7 out of 11 also lost HCV-RNA from both serum and leukocytes [9]. Although Italian scientific literature is rich with controversial epidemiological data obtained in limited populations, the pattern of HCV infection in the whole population overall is poorly defined. In a recent issue of Hepatitis Monthly, La Torre et al. estimated HCV infection trends in Italy during the years 1996-2006 [10]. To evaluate the incidence rates, the authors integrated specific ministerial data (Ministry of Health) with population data from the National Institute of Statistics (ISTAT), a public and independent research organization. A strong reduction was observed in the analyzed years (-12,45%), distributed equally among males (-12,23%) and females (-12,8%). Considering all age groups, the incidence rate decreased from 2.02 to 0.55 per 100,000. This reduction was significant in all age groups [10]. Reported prevalence and incidence rates of HCV infection in the community vary, not only depending on the specificity and sensitivity of the method used, but also according to the study design, geographical origin, and other characteristics of the included populations [4]. Many studies have been carried out in blood donors or in other specific populations (for example intravenous drug users). Scarce data is however available in the general population. The strengths of the study by La Torre et al. lie in the study design, which included the whole population over a long period of follow-up, and in the use of the joinpoint regression method. The latter is useful in determining the occurrence of changes during distinct periods of time in trend data. Thus, it identifies the calendar years in which statistically significant changes in the trends occur, and the annual percentage of change within the identified period. On the other hand, the potential limitations, as reported by the authors, are the poor quality of reporting. In conclusion, studies such as that of La Torre et al. [10], are crucial in increasing our knowledge of worldwide HCV infection rates and to plan health policy strategies.
